# Synergistic effect of graphene oxide and silver nanoparticles as biostimulant improves the postharvest life of cut flower bird of paradise (Strelitzia reginae L.)

**DOI:** 10.3389/fpls.2022.1006168

**Published:** 2022-09-29

**Authors:** Meenakshi Thakur, Anjali Chandel, Shweta Guleria, Vipasha Verma, Raghawendra Kumar, Gurpreet Singh, Anjali Rakwal, Diksha Sharma, Bhavya Bhargava

**Affiliations:** ^1^ Floriculture Laboratory, Agrotechnology Division, Institute of Himalayan Bioresource Technology-Council of Scientific and Industrial Research, Palampur (HP), India; ^2^ Academy of Scientific and Innovative Research, Ghaziabad, Uttar Pradesh, India; ^3^ Biotechnology Division, Institute of Himalayan Bioresource Technology-Council of Scientific and Industrial Research, Palampur (HP), India

**Keywords:** antioxidant, cut flower, enzymes, nanoparticles, senescence, vase life

## Abstract

The bird of paradise (*Strelitzia reginae* L.) is one of the important tropical cut flowers. Generally, flowers like bird of paradise (BOP) grown for the commercial ornamental market must be of high pre and postharvest quality. Thus, to improve the postharvest longevity and increase marketability, the relative efficacy of two different biologically synthesized nanoparticles (NPs) was evaluated. The novel proprietary stimulants were graphene oxide (GO) and silver nanoparticles (SNPs). The NP treatments were applied as a vase (lower concentrations) solutions. Among all the applied treatments, the synergistic effect of GO + SNPs at 1 µL L^−1^ vase solution significantly (*p* =0.05) prolongs the post-harvest life of cut flowers of BOP. Increased vase life over the deionized water (DI) control was associated with better maintenance of relative water uptake, relative fresh weight, suppressed microbial density at stem-end and delay of stem blockage, reduced electrolyte leakage, malondialdehyde (MDA), SOD, and POD activity. In contrast to control, administration of NPs gave better results for all analyzed parameters. Application of biologically synthesized NPs in combination (GO + SNPs at 1 µL L^−1^) extended the vase life of cut flowers by 6 days compared with control flowers, and overall, showed better results than the control. The findings of the studies revealed that the standardized NPs could have more potential in prolonging the postharvest life of cut flowers in BOP. Thus, this technique can be used as a novel postharvest technology for commercial application in cut flowers.

## Graphical Abstract

Exogenous application of NPs increased the postharvest life of bird of paradise.

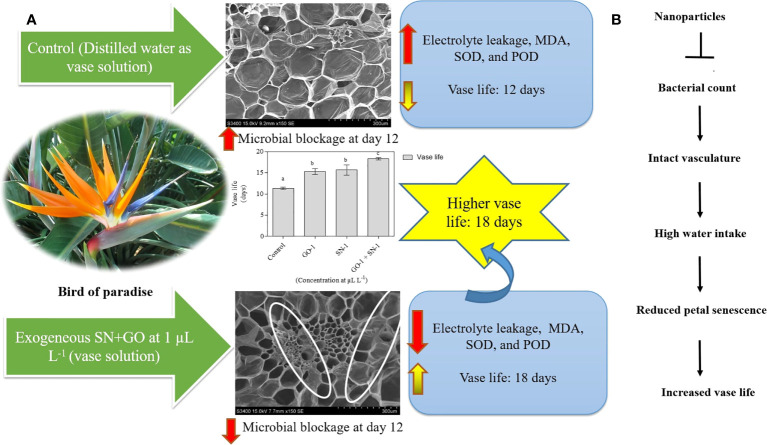

## Introduction

The bird of paradise (*Strelitzia reginae* L.) of the Strelitziaceae family is an evergreen, monocotyledonous, perennial herbaceous flowering plant (Rai et al., 2021). It is commonly referred to as the crane flower, which plays a significant role in floral arrangements due to its exotic specialty, the combination of orange and purple colored flower clusters, and the unique shape of the crested head of a bird (Sane et al., 2020; Pereira et al., 2021). Conventionally, this plant is propagated through seeds and rhizomes and has a long gestation period of about 4–7 years (Navyashree et al., 2017; Sane et al., 2020). Its commercial cultivation occurs in North America, California, Israel, South Africa, Netherlands, Poland, China, and Japan (Pereira et al., 2021). Previous studies reported that the postharvest life of BOP ranged between 6–16 days (d) and 38.5 d (El-Saka et al., 1995). However, more commonly, the postharvest life varies from 6 to 16 d (El-Saka et al., 1995; Bayogan et al., 2008). Growing conditions, harvest stage, different holding conditions, and criteria used for evaluating inflorescence postharvest life, and conventional methods *viz*., cold storage or low temperature, and preservative solutions, could all contribute to extending the vase life of horticulture crops (Kumari et al., 2022; Tayal et al., 2022).

In plants, the water content is absorbed by the xylem vessels of the stem ends, which helps maintain the petal turgor and flower freshness (He et al., 2018). Microbial blockage, on the other hand, reduces the water uptake, resulting in water loss even when the stem is dipped in water and eventually causing a water metabolism imbalance (Jaroenkit and Paull, 2003; He et al., 2018). Additionally, the depletion of carbohydrates also contributes to reducing the postharvest life of cut flowers (Carrillo-Lopez et al., 2016). The longevity of cut flowers can be greatly influenced by the inclusion of various chemicals in the vase solution, such as biocides and growth regulators, which prevent the proliferation of microbes and extend the vase life of cut flowers (Edrisi et al., 2015). Commercial flower preservatives, on the other hand, have yet to be proven to be consistent in the extension of the postharvest life of cut flowers (Jaroenkit and Paull, 2003). Many studies have recently reported that nanoparticles (NPs) can extend the vase life of cut flowers such as gladiolus, roses, BOP, and gerbera by inhibiting bacterial colonization (Hassan et al., 2014; Li et al., 2017; Naing et al., 2017; Azarhoosh et al., 2021).

NPs are new compounds that are widely used for their antimicrobial properties, thermal conductivity, chemical stability, and catalytic activity (Hassan et al., 2014; Yan and Chen, 2019; Sharma et al., 2022; Zafar et al., 2022). Silver nanoparticles (SNPs) have a high surface-to-volume ratio and have a specific ability to get oxidized, which strongly inhibits microbial growth (Basiri et al., 2011; Rafi and Ramezanian, 2013). Furthermore, these NPs interact with the phosphate (DNA), sulfur (proteins), and thiol groups (enzymes) of the biomolecules and interfere with the microbial metabolism, ultimately preventing microbial growth (Prabhu and Poulose, 2012; Yin et al., 2020). Another NP, graphene oxide (GO), is known for excellent antimicrobial properties by inducing the degradation of inner and outer cell membranes of various microbes, which reduces their viability (Xu et al., 2017; He et al., 2018). GO as an effective antimicrobial agent is expected to improve plant growth and extend the life of cut flowers after harvest.

Therefore, the vase life can be extended by placing cut stems in a preservative solution and providing them with a suitable environment for delaying senescence (Langroudi et al., 2019). Because of its global popularity, SNPs and GO were used as novel antimicrobial agents for the longevity of BOP in the current study. The rationale for the study approach is water metabolism in BOP and protection of stem ends from bacterial blockage by using NPs. To date, there has been no report on the use of NPs to extend vase life in BOP. Therefore, the present study hypothesized that use of an optimal dose of SNPs, GO and its synergistic effect can effectively prevent microbial colonization and ensure the proper water uptake through the xylem vessel, which ultimately increases the vase life of cut flowers of BOP.

## Materials and methods

### Plant material

The cut spike of BOP was harvested in October 2021 from the eight-year-old plantation of BOP cultivated under the shade net house of the Floriculture Farm of the Agrotechnology Division, Council of Scientific and Industrial Research-Institute of Himalayan Bioresource Technology, Palampur, Himachal Pradesh, India. These cut spikes were harvested at the commercial stage when the spike had an unopened bract on the upper side and a prominent orange knuckle. Within 1 h, the stems were placed upright in a bucket partially filled with tap water and transported to the laboratory (Liu et al., 2021). Prior to the experiment, stems were uniformly sorted and recut to a length of 50 cm under distilled water to avoid air embolism (Liao et al., 2012).

### Experimental details

The experiment was carried out at a temperature of 20.7–25.2°C with a relative humidity of 46.7–61.9% and a photoperiod of 12 h at an intensity of 15 μM m^−2^s^−1^. Prior to the experiment, the glass vases were surface sterilized with ethanol. To determine the concentrations of NPs, trial screening on phenological studies of cut spikes of BOP was performed by using different concentrations of NPs *viz*., T_1_: Control (distilled water), T_2_: GO at 0.5 µL L^−1^, T_3_: GO at 1 µL L^−1^, T_4_: GO at 3 µL L^−1,^ T_5_: SNP at 0.5 µL L^−1^, T_6_: SNP at 1 µL L^−1^, T_7_: SNP at 3 µL L^−1^ and T_8_: GO + SNPs at 1 µL L^−1^. Based on preliminary observations, the following treatments, *viz.*, T_1_: Control (distilled water); T_2_: GO at 1 µL L^−1^; T_3_: SNP at 1 µL L^−1^ and T_4_: GO + SNPs at 1 µL L^−1^ (a synergistic effect of NPs) were selected and analyzed at 0, 6, 12, and 18 d. Nanoparticles *viz*., SNP and GO solutions were purchased from Sigma Aldrich as silver dispersion nanoparticles, with a 10 nm particle size (TEM), 0.02 mg mL^−1^ in aqueous buffer (Code:1003234773) and graphene oxide, 2 mg mL^−1^, dispersed in water (Code:1003039080). These solutions were freshly prepared before the execution of experiments by dissolving dimethyl sulfoxide (DMSO) with SNP and GO solution. Each cut spike was individually placed in a 500 mL vase solution. To prevent evaporation and microbial contamination of preservative solutions, the tops of the vases were covered with thermocol. The solutions were only prepared and placed in the various vases on the first day, and they were not renewed during the storage period. This experiment was executed in a completely randomized block design. In total, 18 spikes from each treatment in three replications (six spikes in each replication) were taken for the experimental study.

### Water uptake, relative fresh weight, and floret opening

Water uptake was measured at regular intervals (mL) by weighing the vases without flowers and individual flowers separately, then combining the results at the end of the vase life assessment (Li et al., 2012; Liao et al., 2013). After harvest, the initial fresh weight of cut flower stems was measured immediately before placing them in a vase solution. In all the treatments, fresh weight was recorded at 0, 6, 12, and 18 d and the relative fresh weight of each stem was calculated as the percentage on the basis of the initial fresh weight (Singh et al., 2008).

### Flower diameter and vase life

The diameter of each flower was measured at 0, 6, 12, and 18 d using a Vernier calliper. For each treatment, six cut flowers were selected for calculation, and their mean was taken as the final value. The vase life of each flower was determined by the time it took for one-third of its petals to show inward rolling, browning, discoloration, wilt, and loss of turgidity after being placed in various vase solutions (Liao et al., 2013; Lin et al., 2019).

### Microbial analysis

The bacterial isolation was carried out from a 12-d-old spike using the method described by Balestra et al. (2005) and Li et al. (2012), with some modifications. Specifically, the proximal end of the BOP spike was cut with sterile scalpel blades that had been surface sterilized with 70% ethanol (Liu et al., 2021) and then cut up into small pieces. These pieces were placed in sterile tubes with 1 mL of sterile 0.9% normal saline solution (Li et al., 2012). Bacteria were dislodged by using a sterile motor pestle to crush the small pieces. The serial dilution was done with a sterile 0.9% normal saline solution, from which 0.1 mL of liquid extract was spread onto nutrient agar plates and incubated for 24 h at 37°C.

### Analysis of xylem blockage by scanning electron microscopy (SEM)

The existence of bacteria in the xylem vessel at the stem end of the cut flower was analyzed by using scanning electron microscopy (SEM) (JEOL, Japan). The samples of the cut spike from the end were collected and analyzed on day 12 of the vase period. Using fresh surgical blades, 1 cm-long stem-end segments were excised from the base of each spike. The samples were examined using SEM, which works on the principle of focusing a beam of electrons (0.2–20 KeV) from a tungsten filament on the specimen (Thakur and Kumar, 2021). On an aluminum stub, the samples were mounted with double-sided carbon tape. Samples were coated with a thin layer of gold using a sputter-coater at a vacuum of 10 Pa for 10 s to provide electrical conductivity. At a 30 kV accelerating filament voltage, the images were captured at the desired magnification (Thakur and Kumar, 2021).

### Electrolyte leakage

The electrolyte leakage was evaluated by following the procedure given by Danish and Zafar-ul-Hye (2019). Briefly, 12-day-old petals were collected and washed with deionized water and punched using a cork borer having a diameter of 1 cm, in triplicate. Petal pieces of uniform size were immersed in a test tube containing 10 mL of deionized water and incubated for 24 h at 25°C. A pre-calibrated electrical conductivity meter (PC 700, Eutech Instruments, Singapore) was used to determine the solution’s initial electrical conductivity (EC1). The solution was heated in an autoclave at 120°C for 20 min, and conductivity (EC2) was measured again after cooling at room temperature, and electrolyte leakage (EL) was calculated by using the formula: EL = EC1/EC2 × 100 (Kaya et al., 2002).

### Enzymatic activity

The petals were collected on day 12 for enzymatic activity assays.

### Extraction of total soluble proteins and quantification

The tissue of 200 mg was ground in liquid nitrogen and dissolved in 5 mL of 50 mM potassium phosphate buffer at pH 7.8, followed by the addition of 2% polyvinylpolypyrrolidone. The homogenized solution was centrifuged at 8,000×*g* for 20 min and the supernatant was used for superoxide dismutase and peroxidase activity. Protein was quantified according to the protocol given by Bradford (Bradford, 1976). As a standard, bovine serum albumin was used.

### Superoxide dismutase and peroxidase assay

In a 96-well plate, an enzymatic assay for superoxide dismutase (SOD) activity was performed using the nitro blue tetrazolium (NBT) reduction assay as described by Sahoo et al. (2001) for day 12 old petals. In brief, to a final volume of 200 μL, 50 mM potassium phosphate buffer (pH 7.8), 20 μL of total protein, 5.7 × 10^−5^ M NBT, 9.9 × 10^−3^ M methionine, 1.17 × 10^−6^ M riboflavin, and 0.025% Triton X-100 were added. For the assay control, total protein was replaced with an equal volume of 50 mM phosphate buffer (pH 7.8). At room temperature, the reaction mixture was illuminated with white light for 10 min. The absorbance was taken at 560 nm by using a microtiter plate reader (Synergy HT, BioTek, USA) (Chafik et al., 2019; Guleria et al., 2021). SOD activity was measured in units per mg of protein. One unit of the activity of an enzyme is the amount of enzyme required to suppress NBT reduction by 50% at room temperature in 10 min as per the following formula: Percent inhibition = [(*A*_560_ control − *A*_560_ sample)/*A*_560_ control] × 100 (Kumar et al., 2016).

The peroxidase activity was measured by quantifying the amount of purpurogallin formed in 12-d-old petals. The peroxidase activity was carried out using the protocol of Kar and Mishra (1976) with a minor modification. To the 10 µL of crude enzyme, 165 μL of potassium phosphate buffer (50 mM, pH 7.8), 20 μL of pyrogallol (0.5 M), and 5 μL of H_2_O_2_ (0.2 M) were added to a total volume of 200 μL. The amount of purpurogallin formed during the reaction was evaluated by recording absorbance at 420 nm for 5 min. Enzyme activity was measured in unit g^−1^ of fresh weight and calculated using the following formula: (Δ*A*_420_/20 s test sample − Δ*A*_420_/20 s Blank) (volume of the assay) (dilution factor)/(12) (volume of enzyme used) (Kwak et al., 1995; Zhu et al., 2019).

### Lipid peroxidation assay

Lipid peroxidation was measured by quantifying malondialdehyde (MDA) content in 12 d-old petals. A petal sample of 200 mg was homogenized by using 2 mL of 50 mM Tris-Cl, pH 8.0 solution containing 0.5% (w/v) thiobarbituric acid, 20% (v/v) trichloroacetic acid, and 0.25 mL of 175 mM NaCl. The mixture was boiled for 5 min at 100°C, then cooled in an ice bath for 5 min (Dangol et al., 2021). The solution was centrifuged at 14,000×*g* for 5 min at 4°C, and the absorbance of the resulting supernatant was measured at 450, 532, and 600 nm. MDA content (C) was calculated as µmol g^−1^ of fresh weight using the formula: C = 6.45 (*A*_532_ − *A*_600_) − (0.56 *A*_450_).

### Statistical analysis

The results of the experiment were analyzed using the analysis of variance (ANOVA) method. Within each treatment, there were six inflorescences. Here we have presented significant data for GO at 1 µL L^−1^, SNPs at 1 µL L^−1^, and a combination of GO + SNPs at 1 µL L^−1^ at 6, 12, and 18 d. The Waller–Duncan multiple range test was used to compare means, and for statistical analysis, the SAS statistical package (SAS Institute Inc., Cary, NC) was used. The least significant difference test was used to compare means at the 0.05 probability level (Li et al., 2017). The data have been presented as means ± standard errors.

## Results

### Relative water uptake (mL)

**Figure 1:**
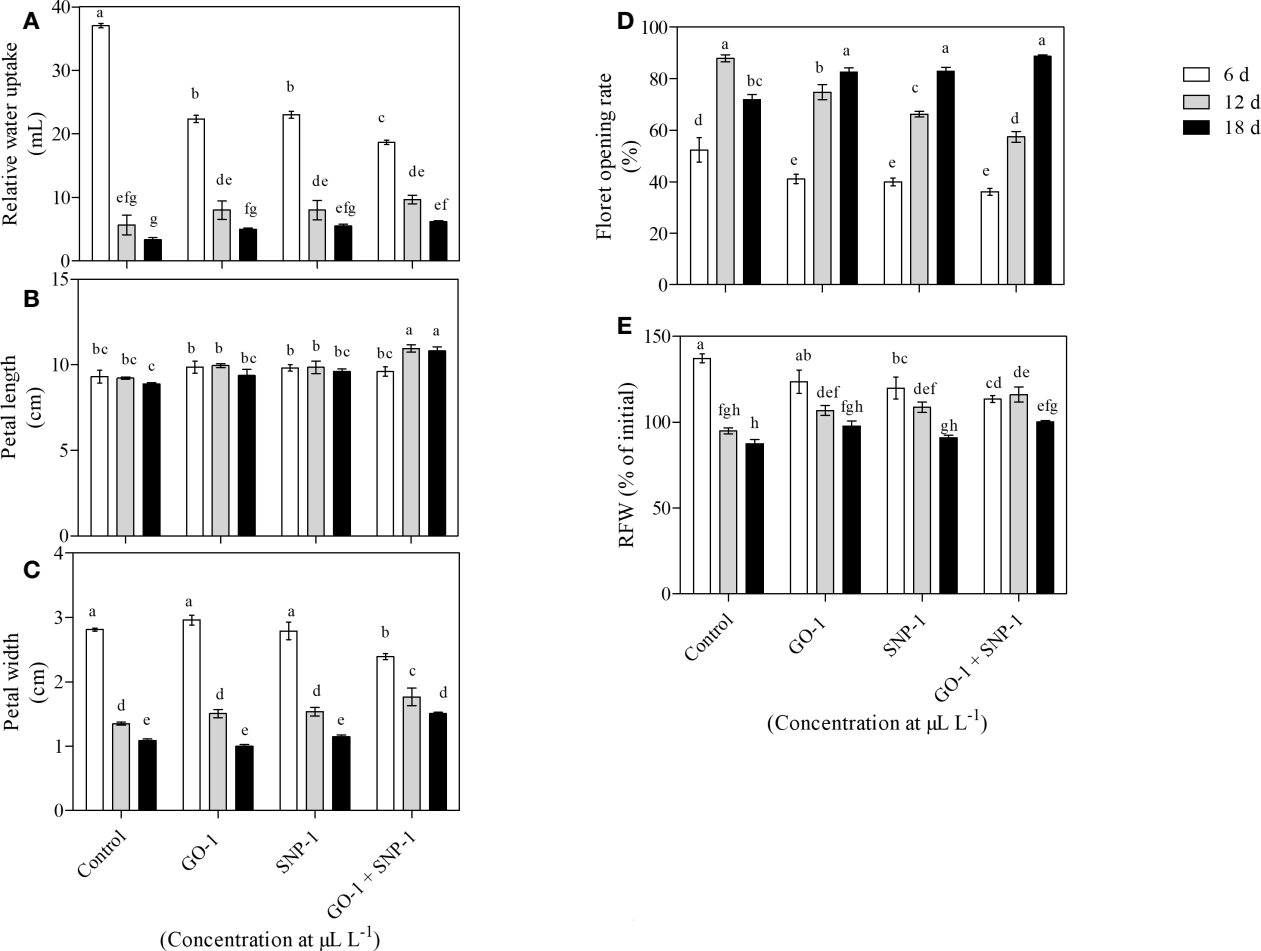
Effect of NPs, *viz*., control, GO, SNP and GO + SNP at 1 µL L^−1^ on various morpho-physiological parameters such as **(A)** relative water uptake, **(B)** petal length, **(C)** petal width, **(D)** floret opening, and **(E)** RFW. Results are shown as mean ± SE of three replicates. Means with different letters are significantly different (Duncan’s multiple range test, *p = 0.05*).

Cut spikes treated with distilled water recorded efficient water uptake (37.0 mL) followed by SNP at 1 µL L^−1^ (23.0 mL) and GO at 1 µL L^−1^ (22.3 mL) along with GO + SNP at 1 µL L^−1^ (18.7 mL). These cut spikes survived until day 6 without showing any signs of senescence (**Figure 1A**). Relative water uptake was reduced significantly on day 12 in all the treatments and reached up to 5.7 to 9.7 mL only. A significant reduction was observed in control and it exhibited petal senescence (**Figure 1A**), whereas significantly higher water uptake was recorded in cut spikes placed in GO + SNP at 1 µL L^−1^ (9.7 mL). Flowers treated with combinations of NPs showed no senescence symptoms until 6 d. Relative water uptake decreased with the increase in the number of days, and was found to be significantly less in all the treatments at 18 d (3.0 to 6.2 mL). While comparing treatments, cut spikes placed under control showed significantly lower water uptake (3.3 mL) as they exhibited senescence on day 12. Relative water uptake was significantly higher in cut spikes placed along with the NPs, i.e., GO + SNP at 1 µL L^−1^ (6.2 mL), which was not statistically different from treated SNP at 1 µL L^−1^ (**Figure 1A**).

### Petal length and petal width (cm)

Petal length and petal width were significantly affected by different vase solutions and the number of days (**Figures 1B, C**). Petal length decreases with an increase in the number of days in all the treatments except for GO + SNP. On day 6, petal length ranged from 9.3 to 9.9 cm. However, no such statistical difference was observed within the treatments. Significantly higher petal length was recorded in the cut spikes placed along with two different NPs, i.e., GO + SN at 1 µL L^−1^ (11.0 cm) compared with control (9.2 cm), while, the latter one was statistically at par with the individual solutions of GO (10.0 cm) and SN (9.9 cm) at 1 µL L^−1^ concentration in 12 d. Petal length on day 18 was reduced significantly in all the vase solutions. However, it was observed significantly higher for cut spikes placed in GO + SNP at 1 µL L^−1^ solution (10.8 cm) compared to the control. Petal length under control (8.9 cm) was not statistically different from the rest of the other treatments (**Figure 1B**).

Petal width decreased significantly with an increase in the number of days, and it was observed to be significantly higher on day 6. Among treatments, the significantly lowest petal width was recorded in cut spikes treated with the combined effect of GO + SNP at 1 µL L^−1^ solution (2.4 cm) (**Figure 1C**). However, the rest of the treatments were statistically at par with each other and showed higher petal width on 6 d. A reduction in petal width was observed in control (1.4 cm) on day 12, while it was significantly higher in GO + SNP at 1 µL L^−1^ solution (1.8 cm). The lowest petal width was observed on day 18 in all the treatments, and it was significantly lower in the absence of treated cut spikes (1.0 cm) as compared with the combination of GO + SNP at 1 µL L^−1^ solution (1.5 cm). Control-treated cut spikes were statistically at par with GO (1.0 cm) and SNP at 1 µL L^−1^ (1.1 cm) (**Figure 1C**).

### Floret opening, relative fresh weight (RFW), and vase life

Floret opening rate was significantly affected by different treatments, with an increase in the number of days (**Figure 1D**). At day 6 floret opening rate was observed significantly lowest in all the cut spikes and when we compared different treatments, it was recorded significantly higher in control (52.4%) followed by GO at 1 µL L^−1^ (41.1%), SNP at 1 µL L^−1^ (39.9%) and GO + SNP at 1 µL L^−1^ (36.1%). Floret opening rate increased significantly on day 12 in all the treatments, which was recorded significantly higher in cut spikes placed in control (87.9%) and lowest with the combinations of NPs, *viz*., GO + SNP at 1 µL L^−1^ (57.4%). On day 18, the floret opening rate starts decreasing significantly in control (71.9%). However, a significant increment was observed in cut spikes treated with NPs alone and in combination with each other. Floret opening rate was recorded significantly higher till 18 d in GO + SNP at 1 µL L^−1^ (88.7%) cut spikes which were not statistically different from GO at 1 µL L^−1^ (82.5%) and SNP at 1 µL L^−1^ (82.9%) treated cut spikes (**Figure 1D**).

Relative fresh weight (RFW) was significantly influenced by different treatments, with an increase in the number of days (**Figure 1E**). On day 6, RFW was observed to be significantly higher in cut spikes placed in control (137.2%) as compared to GO at 1 µL L^−1^ (123.7%), SNP at 1 µL L^−1^ (119.9%) and GO + SNP at 1 µL L^−1^ solution (113.4%). RFW was reduced significantly with an increase in the number of days between 12 and 18 d. Flowers placed in control produced significantly lower RFW. However, the combination of GO + SNP at 1 µL L^−1^ vase solution produced significantly higher RFW (116.1%) at 12 d. The cut spikes treated with GO (106.8%) and SNP at 1 µL L^−1^ solutions (108.7%) were not statistically different from each other, but these treatments produced significantly higher RFW in comparison to control. On day 18, RFW reduced significantly in all the treated cut spikes, while it was observed to be significantly higher in spikes treated with GO + SNP at 1 µL L^−1^ vase solution (100.2%) as compared to other treatments (**Figure 1E**).

**Figure 2:**
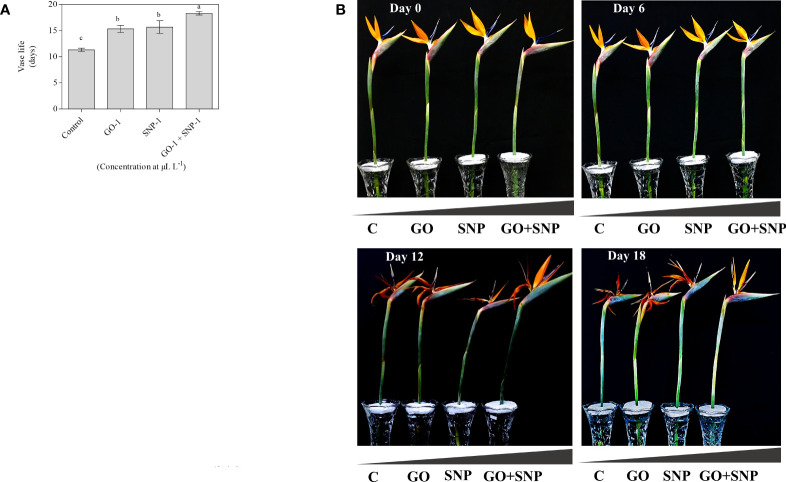
Effect of NPs (C, GO, SNP and GO + SNP at 1 µL L^−1^ on **(A)** vase life and **(B)** vase performance on cut spikes of bird of paradise. Means with different letters are significantly different from each other (Duncan’s multiple range test, p = 0.05).

The vase life of BOP significantly influenced by different treatments of NPs. Plants treated with the combination of GO + SNP at 1 µL L^−1^ produced significantly longer vase life up to day 18 in comparison to control (**Figure 2A**). The cut spikes placed in an individual solution of GO and SNP at 1 µL L^−1^ did not differ from each other for the duration of the vase period up to day 18 (**Figures 2A, B**). Cut spikes of BOP placed in different treatments were captured at 0, 6, 12, and 18 d (**Figure 2B**). This shows the variability of growth in cut spikes of BOP with an increase in the number of days. On day 6, all the cut spikes of BOP placed in different treatments showed a significant increase in the floret opening. On day 12, cut spikes placed under control exhibited different symptoms of senescence and showed more loss of visual quality than other treatments. Spikes placed in GO + SNP at 1 µL L^−1^ solution produced a significantly higher floret opening rate till day 18, as presented in **Figure 2B**.

### SEM observation and microbial analysis

**Figure 3:**
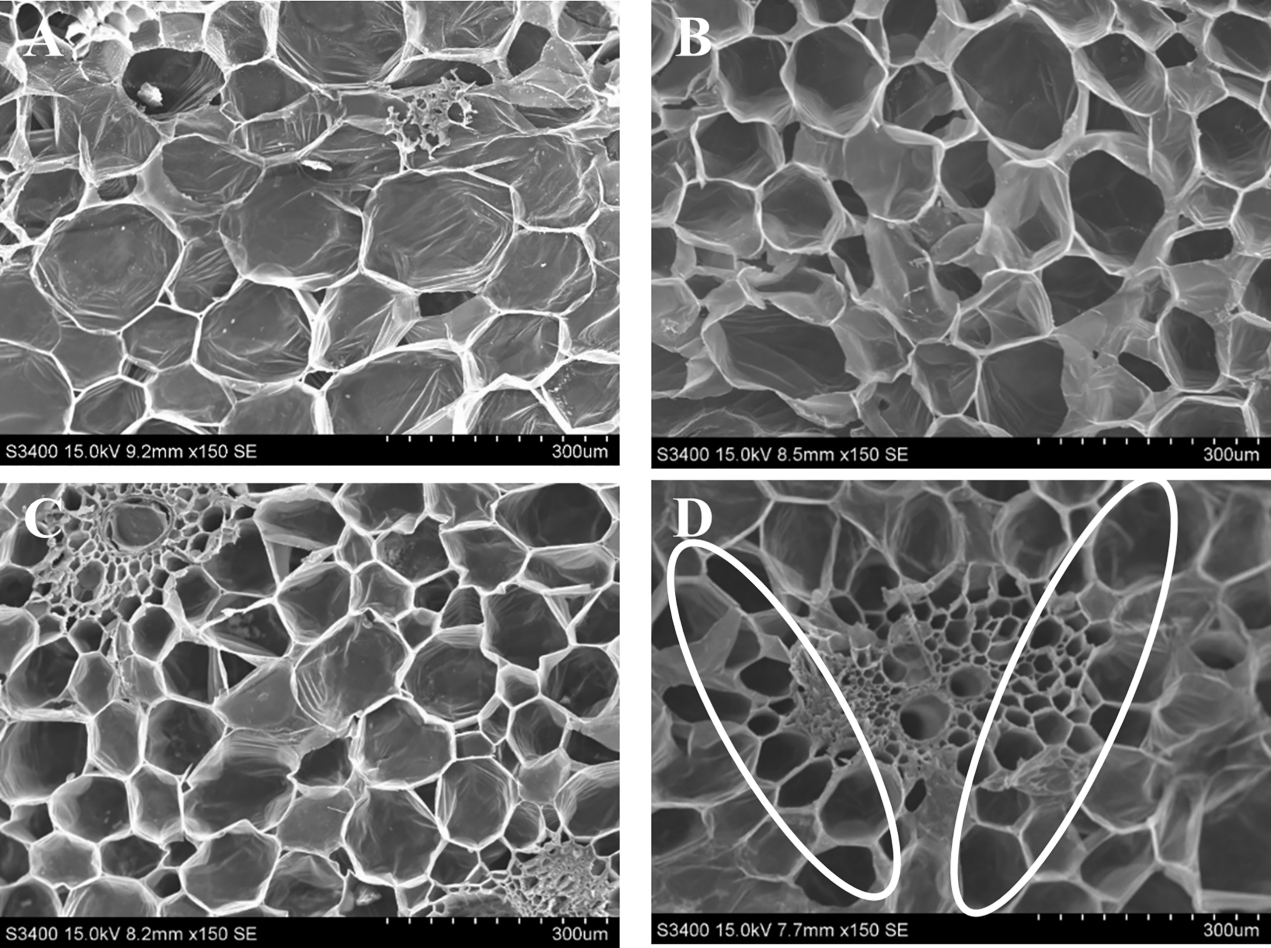
SEM characterization of stem ends of bird of paradise treated with **(A)** Control, **(B)** GO at 1 µL L^−1,^ **(C)** SNP at 1 µL L^−1^, and **(D)** GO + SNP at 1 µL L^−1^ at 12 **(D)** Scale bars: 300 μm.

The relationship between microbes and the longevity of cut spike is shown by the density of bacteria present in the stem end segments. The SEM images in **Figure 3** showed that on day 12, the stem ends of cut spikes were completely filled with a high density of bacteria under control conditions, which showed a reduction in the water-uptake channel. The cut spikes treated with GO at 1 µL L^−1^ solution reduced the bacterial colonization, followed by SNP at 1 µL L^−1^ and GO + SNP at 1 µL L^−1^ solution. In comparison to all the treatments, lower bacterial density was recorded with the combined solution of GO + SNP at 1 µL L^−1^ (**Figure 3**).

**Figure 4:**
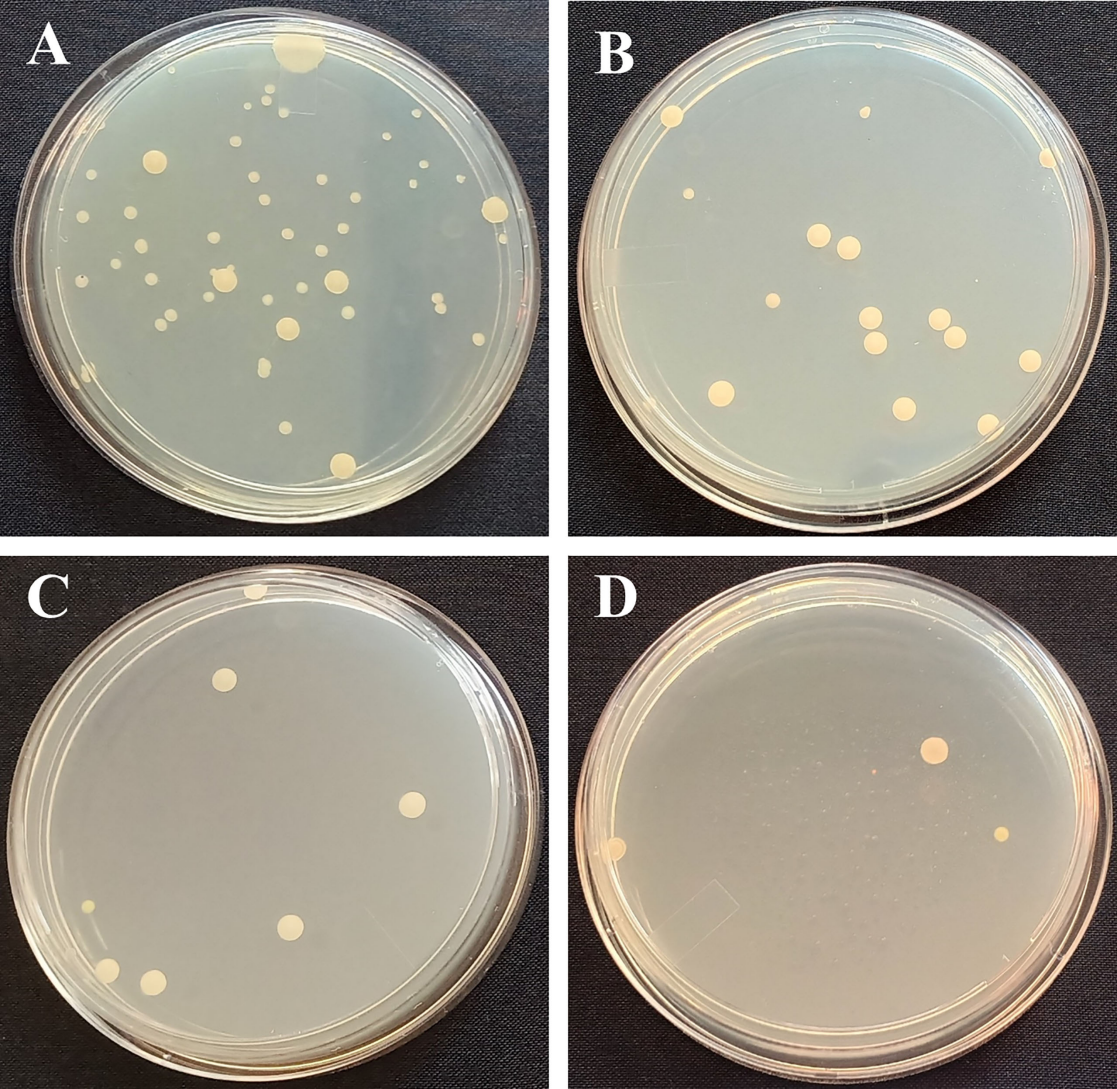
Photographs of petri dishes of **(A)** Control, **(B)** GO at 1 µL L^−1,^ **(C)** SNP 1 µL L^−1^, and **(D)** GO + SNP 1 µL L^−1^.

Microbes in the GO, SNP, and GO + SNP solutions were counted on the same day of SEM observation to further prove the antimicrobial activity of NPs. In the current experiment, we analyzed bacterial density at day 12 when most of the petals were opened and few were in senescence due to the reduction of water uptake. The distilled water group (control) had a significantly higher number of bacteria, with 37 × 10^−5^ colony forming units (cfu) per mL (about equal to bacteria per mL) (**Figure 4**). As for the cut spikes treated with GO at 1 µL L^−1^ concentration, the bacterial density was recorded up to 14 × 10^-5^ cfu mL^−1^ (**Figure 4**). The spikes treated with SNP at 1 µL L^−1^ solution produced a bacterial density of 7 × 10^−5^ cfu mL^−1^. The stem ends dipped under the combination of GO + SNP at 1 µL L^−1^ had a lower bacterial density (4 × 10^-5^ cfu mL^−1^) (**Figure 4**). Based on the phenotype, we have analyzed that only a single type of bacteria was found in the cut spike of BOP.

### Electrolyte leakage measurement

**Figure 5:**
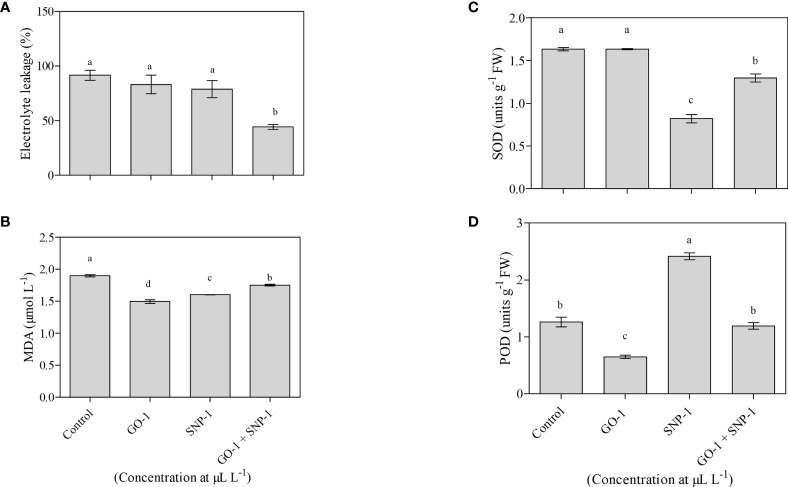
Effect of NPs **(A)** Control, **(B)** GO at 1 µL L^−1,^ **(C)** SNP 1 µL L^−1^, and **(D)** GO + SNP 1 µL L^−1^ on **(A)** Electrolyte leakage, **(B)** MDA, **(C)** SOD activity, and **(D)** POD activity in the petals of bird of paradise cut flowers. The samples were collected at day 12. Each data point indicates mean of three independent biological replicates. Error bars indicate SE of mean. Means with different letters are significantly different (Duncan’s multiple range test, *p = 0.05*).

Changes in electrolyte leakage were observed in petals corresponding to changes in membrane permeability. The least ion leakage was observed in florets kept in a solution containing GO at 1 µL L^−1^ and SNP at 1 µL L^−1^ solution. This was significantly (*p* = 0.05) lower than the control and other treatments (**Figure 5A**), confirming the integrity of the cellular membrane.

### Malondialdehyde content (MDA)

A significant (*p* = 0.05) accumulation of MDA was observed in the untreated flower stalk (control), which accounts for more lipid peroxidation. However, treatment with GO at 1 µL L^−1^ and SNP at 1 µL L^−1^, both separately and in combination, significantly (*p* = 0.05) reduced the accumulation of MDA when compared with untreated cut spikes (**Figure 5B**).

### Superoxide dismutase (SOD) and peroxidase assay (POD)

SOD activity decreases significantly (*p* = 0.05) on day 12 for florets kept in SNP at 1 µL L^−1^, solitary and in combination with GO at 1 µL L^−1^, compared to the control. While there was no significant (*p* = 0.05) difference in SOD activity in florets kept in GO at 1 µL L^−1^ vase solution as compared to the control (**Figure 5C**). POD activity increases significantly (*p* = 0.05) in day 12 florets kept in SNP at 1 µL L^−1^ solution in comparison to the control. However, when florets were kept in GO at 1 µL L^−1^ solution alone or in combination with SNP at 1 µL L^−1^ solution, a significant (*p* = 0.05) decrease was observed, while no significant (*p* = 0.05) effect on POD activity was observed, respectively, as compared with cut spikes placed in distilled water (**Figure 5D**).

## Discussion

Multi-colored BOP flowers are very showy, thus have a great global demand as cut flower. Cut spikes usually suffer water stress when they are detached from the mother plant (Halevy and Mayak, 1981), which results in irregular floret opening, quick loss of chlorophyll, proteolysis, and enhanced membrane permeability, and premature wilting premature wilting, which ultimately reduces the postharvest life of flowers. The senescence of cut spike cannot be eliminated. However, it can be regulated by various postharvest techniques (van Doorn, 2012; Rabiza-Swider et al., 2020). Therefore, microbial blockage at the stem end is considered to be the major key factor for reducing the vase life of cut flowers. In addition, proper water uptake, transport, and prevention of microbial blockage are effectively utilized for the development of buds to bloom and to boost the postharvest life of cut flowers (Solgi et al., 2009; Lu et al., 2010; Naing and Kim, 2020). The use of toxic chemicals results in environmental pollution, which will eventually affect human health. NPs at low concentration have been reported to be a safe biocide capable of extending the postharvest life of cut flowers (Zhao et al., 2018; Rabiza-Swider et al., 2020). As a result, the current study was carried out to assess the effect of NPs in increasing the postharvest life of cut spikes of the BOP. NPs are made up of small particles that appear to extend the postharvest life of cut flowers by inhibiting their antibacterial properties (Williamson et al., 2011; Liu et al., 2012).

Water uptake is a crucial factor in improving the vase life and quality of cut flowers (Reid and Evans, 1985; Naing et al., 2017) and depends on water storage, hydraulic conductivity, salinity, and water potential (Solgi et al., 2009; Naing and Kim, 2020). The proliferation of bacterial growth in the xylem vessels is the primary cause of water transport to cut flowers, so bacterial growth in the xylem vessels can obstruct water uptake in the vase (Park et al., 2017; Xia et al., 2017; Naing and Kim, 2020). The current applications of NPs have demonstrated stronger antimicrobial properties that extend the postharvest life of cut flowers, but these studies have not been widely popularized (Ahmed et al., 2016). SNPs and GO also interact with cytoplasmic and nucleic acid components, inhibiting respiratory chain enzymes and interfering with membrane permeability (Lok et al., 2007; He et al., 2018). In the current study, water uptake increased slowly but steadily up to day 18 when cut spikes were placed in combination with GO + SNP at 1 µL L^−1^ solution (**Figure 1A**). The application of NP mostly inhibits gram-positive bacteria and bacterial DNA replication, resulting in increased water absorption in cut flowers (Sondi and Salopek-Sondi, 2004). NPs alone showed significant variation for water uptake in comparison to control. However, the synergistic effect of GO + SNPs showed significant variations. The microbes were effectively killed by the concentration of GO, SNP, and GO + SNP at 1 µL L^−1^ which resulted in clear xylem vessels for water uptake. Relative water uptake decreases with an increase in the number of days in control, which could be attributed to the blockage of water-conducting tissues, particularly xylem vessels, by the accumulation of microbes (bacteria) (van Doorn, 2012). Previous studies reported similar findings on the positive effect of NPs on cut flowers, including carnations, cut roses, lisianthus, tuberose, gladiolus, and lily (Hatami et al., 2013; Bahremand et al., 2014; Hajizadeh-Oghaz et al., 2016; Park et al., 2017; He et al., 2018; Lin et al., 2019; Maity et al., 2019; Naing and Kim, 2020). Besides these studies, no synergistic effect has been reported yet with the combined applications of two different NPs like GO and SNPs. In current studies, petal length and width significantly increased slowly but steadily after day 6 to day 12 when cut spikes were treated with the individual NPs. However, combined GO + SNPs at 1 µL L^−1^ showed the highest petal length and width (**Figures 1B, C**). This could be due to the proper uptake of water (GO + SNPs), which is translocated to the petals, resulting in carbohydrate accumulation and increased petal length and width overall (Rabiza-Swider et al., 2020).

Exposure of cut spikes under control conditions inhibited the floret opening percentage after day 12 (**Figure 1D**), resulting in premature wilting of petals. However, the combination of GO and SNP promoted the flower opening (**Figure 1D**) and it maintained high RFW during the vase period, which ultimately increased the postharvest life of BOP cut flowers till 18 d. Moreover, the GO and SNPs alone had similar effects in enhancing the flower opening, while the synergistic effect of both these NPs was more pronounced. It might be because NP accumulated in stem-ends, and transported up to the receptacle, calyx, and petal tissues (Liu et al., 2018). These findings were synonymous with the findings of Lu et al. (2010) in cut rose flowers. Initially, there was a significant increase in RFW till day 6, after that it started decreasing in all the treatments during the vase life of BOP (**Figure 1E**). RFW and rate of senescence were reduced comparatively with an increase in the number of days in cut spikes treated under control conditions (**Figure 1E**). This might be because of lesser absorption of water uptake, increased respiration rate and ion leakage from petals lead to the damage cell membrane (Singh and Jegadheesan, 2003). Similar findings were reported in petunias, daylilys, roses, and gladiolus (Bieleski and Reid, 1992; Ezhilmathi et al., 2007; Seyf et al., 2012; Ha et al., 2019). Studies reported that NPs treatment in cut roses, gerberas, anthuriums, and carnations increased water uptake rate and RFW by limiting bacterial growth, transpiration rate, and stomatal conductance (Ansari et al., 2011; Rafi and Ramezanian, 2013; Liu et al., 2014; Abdel-Kader et al., 2017; Amin, 2017).

The relationship between flower senescence and protein degradation has been observed in several cut flowers, including carnation, sword lily, daylily, and dendrobium (Vierstra, 1996; Stephenson and Rubinstein, 1998; Sugawara et al., 2002; Azeez et al., 2007; Lerslerwong et al., 2009; Rabiza-Swider et al., 2020). Biofilms play a crucial role in creating a protective microhabitat against environmental stress (Ratnayake et al., 2012; Sharma et al., 2022). The accumulation of MDA is an indicator of the free radical induced peroxidation of the cell membrane, which impairs the function of the lipid membrane (Leverentz et al., 2002; Irfan et al., 2021; Sharma et al., 2021; Kapoor et al., 2022). In current findings, MDA decreases with NP treatments on day 12 (**Figure 5B**), which extends vase life over the control group. Antioxidant enzyme activity is thought to play an important role in cellular defense against oxidative stress (Sevillano et al., 2009; Hassan et al., 2014; Khan et al., 2021). Their activity increases in the presence of high levels of free radicals (Nair and Chung, 2015). Cut spikes treated with GO alone and in combination with GO + SNP at 1 µL L^−1^ had lower levels of SOD and POD activities, which was most likely due to less oxidative stress experienced by cut flowers on day 12 (**Figures 5C, D**). The extended vase life with NPs is associated with the increment of the absorption of relative water uptake (**Figure 1A**), improved water balance, reduction in electrolyte leakage (**Figure 5A**), and integrity of the phospholipid membrane (**Figure 5B**), which inhibited the progress of senescence and resulted in the extension of vase life (Seyf et al., 2012). These results are accompanied by the effect of NPs as an antimicrobial agent, thus increasing the relative water uptake, petal length, width, and floret opening, which increases the RFW and extends the postharvest life of cut flowers of BOP as compared with control (**Figure 2**). The combined application of NPs as preservative vase solutions for cut flower longevity has not been studied yet, thus the current findings are reported for the first time with the synergistic effect of GO + SNP on post-harvest life of cut flowers. Our results confirm the efficacy of SNP in extending the vase-life of BOP cut flowers. NPs are potent ethylene blockers, which may have an additive effect in extending the postharvest life of cut flowers such as cut lily, rose, and carnation (Kim et al., 2005; He et al., 2018; Liu et al., 2018; Lin et al., 2019).

Microbial growth at the stem end is the primary cause of xylem vessel blockage, which prevents water uptake and, as a result, reduces cut flower longevity (Balestra et al., 2005). Stem blockage initially occurs at the stem ends, up to 2 cm (van Doorn and Vaslier, 2002). The NP has antimicrobial properties which have been proved to conserve the postharvest life of cut flowers (Naing and Kim, 2020; Liu et al., 2021). As per SEM observations, cut spikes treated with NPs alone or in combination reduced the density of bacteria in the vase solution (**Figure 3**), which probably improved the water uptake, floret opening, and RFW, resulting in an increased postharvest life of BOP as compared with the cut spikes placed in distilled water. After exposure of treatments to cut spikes, NPs not only inhibit the microbial growth at stem-ends (**Figure 4**) but also enters the xylem to inhibit the bacterial proliferation, and are translocated to flower parts, *viz*., petals and gynoecium leading to a higher uptake of water, floret opening, and RFW which ultimately increase the longevity of cut flowers (Hassan et al., 2014; Naing et al., 2017). Similarly, the reduction of vase solution uptake was related to bacterial density in the stem-ends of cut roses (Li et al., 2012). Furthermore, several studies have reported that NPs have a bactericidal effect, which has an impact on the ability of the cut flower to absorb water (Rai et al., 2009; Lu et al., 2010). In the current studies, bacterial density was observed to be significantly higher in control as presented by SEM images (**Figure 3**), which probably caused microbial blockage at the stem end, resulting in water deficit and inhibiting the vase life of cut flowers (van Doorn and De Witte, 1997). Due to reduced bacterial populations in solutions of NPs, there was a significant increase in the vase life of lily, tuberose, chrysanthemum, and tulip (Nemati et al., 2013; Bahremand et al., 2014; Byczynska and Salachna, 2017). GO has effective antimicrobial properties as it can attach to the cell membranes and penetrate bacteria cells by disrupting the metabolic processes leading to reduce the microbial viability (Tu et al., 2013). However, no such studies have been reported in most of the cut flower crops with the GO and its effectiveness depends on the dosage since excessive concentration clogged vascular tissues and induced oxidative stress that accelerated flower senescence.

## Conclusion

NPs were evaluated as a novel antimicrobial agent for increasing the postharvest life of cut flowers after harvest. In the current study, cut spikes of BOP were treated with various concentrations of NPs and characterized by using various parameters, demonstrating that NPs could extend the vase life of BOP. Cut spikes treated with NPs effectively prevented microbial proliferation, increased the relative water uptake, and led to improve the water relation of cut spikes of the BOP. The synergistic effect of GO + SNP at 1 µL L^−1^ was most effective in preventing the microbial density, increasing relative water uptake, floret opening, RFW, preserving proteins, decreasing MDA accumulation, and boosting the antioxidant enzyme activity. Therefore, the combined application of GO + SNPs can be recommended as the most appropriate vase solution to extend the postharvest life of cut flowers of BOP up to day 18 due to its excellent antimicrobial property, which has great potential in the agriculture sector. Although this is the first study we have reported of cut flowers of BOP, the developed technology could be routinely used to enhance the postharvest life of cut flowers in the commercial sector worldwide. Thus, more future studies are required with appropriate controlled conditions, different concentrations, and combinations with other chemicals and sugars to establish the superiority of NPs.

## Data availability statement

The original contributions presented in the study are included in the article/Supplementary Material. Further inquiries can be directed to the corresponding author.

## Author contributions

MT initiated experimental planning, execution, statistical analysis, data compilation, data curation, data presentation literature search and manuscript writing. AC helped to execute experiments, data observation, SEM observation. SG contributed to biochemical analysis and manuscript writing. VV edit the manuscript and data presentation. RK and DS helped in microbial analysis. AR and GS helped in SEM analysis, data recording and data compilation. BB supervised the research, funding acquisition, project administration, and manuscript editing. All authors contributed to the article and approved the submitted version.

## Funding

The project was funded by the Council of Scientific and Industrial Research (CSIR), Government of India, under CSIR-Floriculture Mission (Project Number: HCP-0037).

## Acknowledgments

The authors are grateful to the Director, CSIR-IHBT, Palampur, (HP), India for providing necessary facilities during study. We are very thankful to Dr. Amitabha Acharya and Mr. Akib Iqbal Dar for helping in the measurement of zeta potential of nanoparticles. Mr. Vikas and Mr. Balwant Raj are also acknowledged for their technical support. This is CSIR-IHBT publication number 5146.

## Conflict of interest

The authors declare that the research was conducted in the absence of any commercial or financial relationships that could be construed as a potential conflict of interest.

## Publisher’s note

All claims expressed in this article are solely those of the authors and do not necessarily represent those of their affiliated organizations, or those of the publisher, the editors and the reviewers. Any product that may be evaluated in this article, or claim that may be made by its manufacturer, is not guaranteed or endorsed by the publisher.
